# FMISO accumulation in tumor is dependent on glutathione conjugation capacity in addition to hypoxic state

**DOI:** 10.1007/s12149-017-1189-9

**Published:** 2017-07-10

**Authors:** Yukiko Masaki, Yoichi Shimizu, Takeshi Yoshioka, Ken-ichi Nishijima, Songji Zhao, Kenichi Higashino, Yoshito Numata, Nagara Tamaki, Yuji Kuge

**Affiliations:** 1 Shionogi Innovation Center for Drug Discovery, Discovery Research Laboratory for Innovative Frontier Medicines, Shionogi & Co., Ltd., Sapporo, 001-0021 Japan; 2 Kyoto University Hospital, 54 Kawaharacho, Shogoin, Sakyo-ku, Kyoto, 606-8507 Japan; 3 Faculty of Pharmaceutical Sciences, Hokkaido University, Sapporo, 060-0812 Japan; 4 Central Institute of Isotope Science, Hokkaido University, Sapporo, 060-0815 Japan; 5 Graduate School of Medicine, Hokkaido University, Sapporo, 060-8638 Japan

**Keywords:** FMISO, Glutathione, Hypoxia, Imaging mass spectrometry, Molecular imaging

## Abstract

**Objective:**

^18^F-fluoromisonidazole (FMISO), a well-known PET imaging probe for diagnosis of hypoxia, is believed to accumulate in hypoxic cells via covalent binding with macromolecules after reduction of the nitro group. Previously, we showed the majority of ^18^F-FMISO was incorporated into low-molecular-weight metabolites in hypoxic tumors, and the glutathione conjugate of reduced FMISO (amino-FMISO-GS) distributed in the tumor hypoxic regions as revealed by imaging mass spectrometry (IMS). The present study was conducted to clarify whether FMISO is metabolized to amino-FMISO-GS within tumor cells and how amino-FMISO-GS contributes to FMISO accumulation in hypoxic cells. We also evaluated the relationship between FMISO accumulation and the glutathione conjugation-related factors in the cells.

**Methods:**

Tumor cells (FaDu, LOVO, and T24) were treated with ^18^F-FMISO and incubated under normoxic or hypoxic conditions for 4 h. The FMISO metabolites were analyzed with LC–ESI–MS. Several glutathione conjugation-related factors of tumor cells were evaluated in vitro. FaDu tumor-bearing mice were intravenously injected with ^18^F-FMISO and the tumors were excised at 4 h post-injection. Autoradiography, IMS and histologic studies were performed.

**Results:**

Amino-FMISO-GS was the main contributor to FMISO incorporated in hypoxic FaDu cells in vitro and in vivo. Total FMISO uptake levels and amino-FMISO-GS levels were highest in FaDu, followed by LOVO, and then T24 (total uptake: 0.851 ± 0.009 (FaDu), 0.617 ± 0.021 (LOVO) and 0.167 ± 0.006 (T24) % dose/mg protein; amino-FMISO-GS: 0.502 ± 0.035 (FaDu), 0.158 ± 0.013 (LOVO), and 0.007 ± 0.001 (T24) % dose/mg protein). The glutathione level of FaDu was significantly higher than those of LOVO and T24. The enzyme activity of glutathione-*S*-transferase catalyzing the glutathione conjugation reaction in FaDu was similar levels to that in LOVO, and was higher than that in T24. Quantitative RT-PCR analysis revealed that the expression levels of efflux transporters of the glutathione conjugate (multidrug resistance-associated protein 1) were lowest in FaDu, followed by LOVO, and then T24.

**Conclusions:**

FMISO accumulates in hypoxic cells through reductive metabolism followed by glutathione conjugation. We illustrated the possibility that increased production and decreased excretion of amino-FMISO-GS contribute to FMISO accumulation in tumor cells under hypoxic conditions.

**Electronic supplementary material:**

The online version of this article (doi:10.1007/s12149-017-1189-9) contains supplementary material, which is available to authorized users.

## Introduction

Hypoxia, or low oxygen concentration, in tumors plays a significant role in tumor progression and angiogenesis, and is associated with cancer resistance towards radiotherapy and chemotherapy [1, 2]. Therefore, techniques for monitoring hypoxic states in tumor tissue non-invasively could prove very valuable, because such techniques would provide useful information for determining optimal therapeutic strategies and would allow more individualized cancer treatment.

**Fig. 1:**
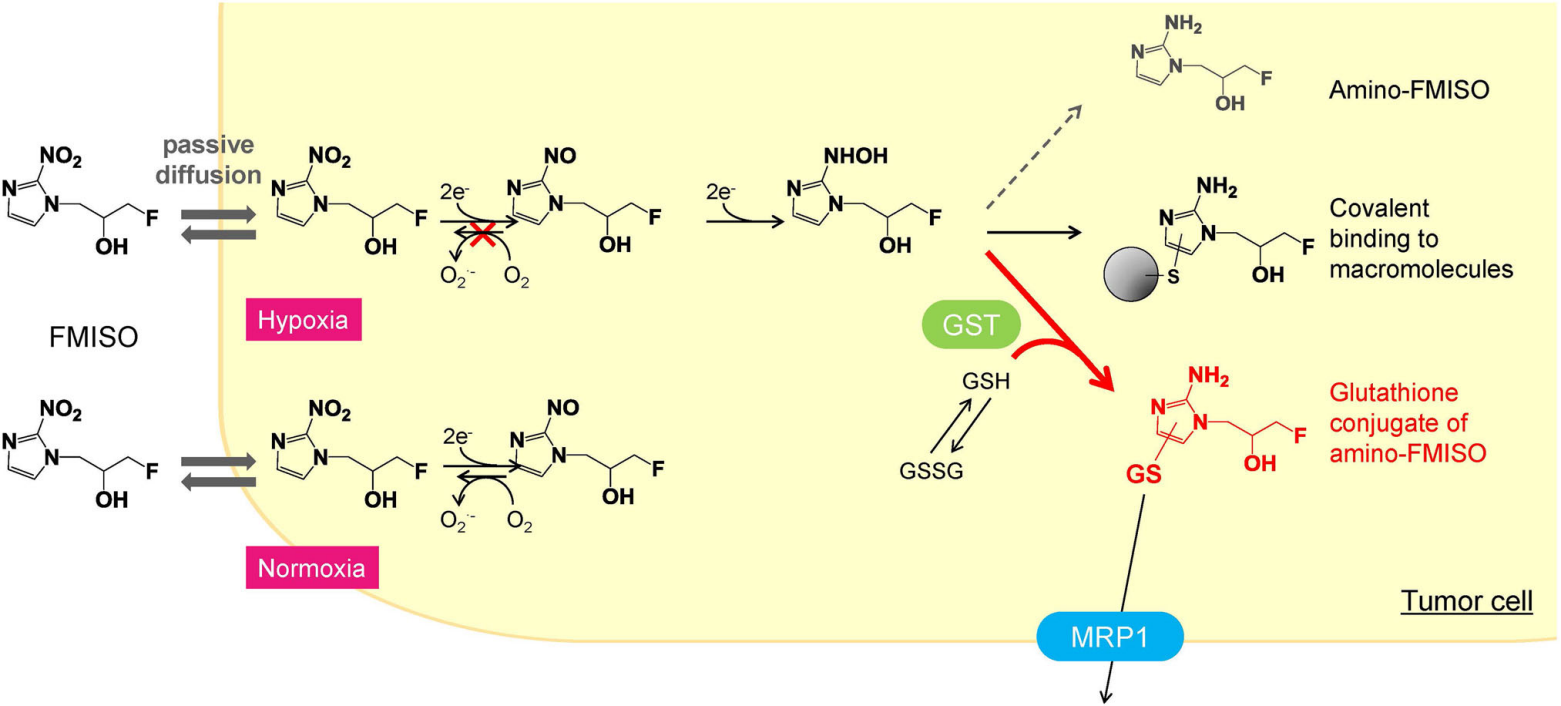
Proposed mechanism of the reduction and accumulation of FMISO in hypoxic cells. The glutathione conjugate in hypoxic cells is the primary contributor to FMISO incorporated in hypoxic cells

Positron emission tomography (PET) with ^18^F-fluoromisonidazole (FMISO), an ^18^F-labeled 2-nitroimidazole derivative, is the most widely used hypoxia imaging technique in clinical diagnosis [3, 4]. FMISO is considered to accumulate in tumor hypoxic regions via covalent binding to macromolecules following reduction of its nitro group (Fig. 1) [5], thereby facilitating the imaging of hypoxic cells. However, this accumulation mechanism remains unclear, since nuclear imaging techniques can provide only the distribution of the radioisotope but no chemical information.

Imaging mass spectrometry (IMS) is a novel imaging technique that can be used to directly visualize the distribution of molecules in tissue sections [6]. In recent years, this technique has been used to investigate the distribution of a wide variety of molecules such as peptides, lipids, drugs, and endogenous metabolites [7–10]. Because it is based on the mass spectrometric detection technique, IMS can distinguish the distribution of multiple molecules in a single measurement without the use of specialized probes. These characteristics enable IMS to evaluate the distribution of specific drug-derived metabolites.

Recently, we investigated the ^18^F-FMISO metabolite specifically found in the hypoxic regions of tumor tissues, using a combination of IMS and radioisotope analysis [11, 12]. In that study, we showed that an unknown low-molecular-weight metabolite exhibited a similar distribution pattern to that of the radioactivity distribution observed for the predicted high-molecular-weight FMISO metabolite complexes, and this metabolite was identified to be the glutathione conjugate of reduced FMISO (amino-FMISO-GS). These results suggested the possibility that amino-FMISO-GS may be the primary contributor to the radioactivity observed in ^18^F-FMISO-PET images, in contrast to the conventional view of FMISO incorporation mainly through covalent binding to macromolecules [13]. However, it still remains unclear whether amino-FMISO-GS is actually produced within hypoxic tumor cells, and how much amount of this metabolite is generated. To clarify them, we first performed the in vitro cellular uptake study and ex vivo metabolite analysis study. We also compared the accumulation amount of FMISO in some cell lines and evaluated the expression and the activity of the biomolecules related to glutathione conjugation in those cells.

## Materials and methods

### Chemicals and reagents

All chemicals were commercially available and of the highest available purity. ^18^F-FMISO was synthesized as previously described [14].

### Cellular uptake study

FaDu human head and neck cancer cells, LOVO colon carcinoma cells, and T24 bladder carcinoma cells (American Type Culture Collection, Manassas, VA, USA) were maintained in Eagle’s Minimum Essential Medium (Sigma-Aldrich, St Louis, MO, USA), Nutrient Mixture F-12 Ham Medium (Sigma-Aldrich) and McCoy’s 5A Medium (Sigma-Aldrich), respectively, supplemented with 10% fetal bovine serum and penicillin (100 U/mL)-streptomycin (100 µg/mL) at 37 °C in a humidified atmosphere of 95% air and 5% CO_2_ (normoxic condition) in 6-well flat bottom plates (Corning Costar, Corning, NY, USA). Cells were pre-incubated for 18 h either under normoxic conditions at 37 °C in a humidified atmosphere containing 5% CO_2_, or under hypoxia conditions at reduced oxygen levels (1% v/v) in an InvivO_2_ 300 incubator (Baker Ruskinn, Ltd., Sanford, ME, USA). ^18^F-FMISO (2.5 MBq, 250 nM/well) was added and the cells were incubated under normoxic or hypoxic conditions. At 4 h post-incubation, the cells were washed three times with phosphate-buffered saline (PBS), suspended in methanol, and then centrifuged. The radioactivity in the supernatants (low-molecular-weight fraction) and the pellets (macromolecule-bound fractions) was measured with a gamma counter (Wizard 3″ 1480 Automatic Gamma Counter; PerkinElmer, Inc., Waltham, MA, USA). The pellets were lysed with 0.2 N NaOH and the protein concentrations of the cell lysates were measured with the bicinchoninic acid (BCA) assay. Cellular uptake study was performed in quadruplicate.

### GSH/GSSG-Glo assay

The reduced glutathione (GSH) and oxidized glutathione (GSSG) levels in the FaDu, LOVO, and T24 cells were measured with a GSH/GSSG-Glo Assay kit (Promega, Madison, WI, USA). In brief, the cells were suspended in PBS and then separated into two aliquots (assay sample for total glutathione fraction and assay sample for GSSG). The assay sample for GSSG and the assay sample for total glutathione were mixed with passive lysis buffer containing luciferin-NT with or without *N*-ethylmaleimide, respectively. The acquired lysates were incubated with Luciferin Generation Reagent at room temperature for 30 min followed by the addition of the Luciferin Detection Reagent. The luciferase reaction was measured in terms of luminescent signal with the Infinite 200 PRO microplate reader (Tecan, Mannedorf, Switzerland). The samples were treated with 0.2 N NaOH and the protein concentrations of the cell lysates were measured with the BCA assay. The GSH and GSSG levels obtained were normalized to the total protein concentrations determined with the BCA assay.

### GST activity assay

GST enzyme activity levels within the FaDu, LOVO, and T24 cell supernatants were measured with the Glutathione-*S*-Transferase Assay Kit (Sigma-Aldrich). In brief, the cells were lysed with 100 mM potassium phosphate (pH 7.0) containing 2 mM EDTA and then centrifuged (1000×*g*, 15 min, 4 °C). The supernatants were mixed with PBS, 200 mM GSH, and 100 mM 1-chloro-2,4-dinitrobenzene (CDNB). The reaction was measured in terms of an increase in the absorbance signal at 340 nm of the reaction product, i.e., the glutathione conjugate of CDNB, using the Infinite 200 PRO microplate reader. The rate of increase in the absorption is directly proportional to the GST activity level in the sample. The samples were lysed with 0.2 N NaOH and total protein concentrations were measured with the BCA assay.

### RNA extraction and real-time PCR

FaDu, LOVO, and T24 cells were cultured at reduced oxygen levels (1% v/v) in the InvivO_2_ 300 for 18 h. After incubation, total RNA was isolated with the RNeasy Mini Kit (Qiagen, Hilden, Germany) according to the manufacturer’s protocols. Reverse transcription was performed with the ReverTra Ace qPCR RT Master Mix (Toyobo Co., Ltd., Osaka, Japan) for RT-PCR on the PCR Thermal Cycler Dice (Takara Bio Inc., Otsu, Japan). Real-time PCR was performed with the SYBR Green Real-time PCR Master Mix (Toyobo Co., Ltd.) on the 7500 Real-time PCR system (Life Technologies, Carlsbad, CA, USA). The expression level of each mRNA was normalized to that of the β-actin mRNA level in each sample. The primer sets used in this study are described in Supplemental Table 1.

### Tumor xenograft model

Nine-week-old male BALB/c athymic nude mice (Japan SLC, Inc., Hamamatsu, Japan) were housed under a 12-h light/12-h dark cycle with food and water supplied ad libitum. All experimental protocols were approved by the Laboratory Animal Care and Use Committee of Hokkaido University and performed in accordance with the Guidelines for Animal Experiments at the Graduate School of Medicine, Hokkaido University. FaDu or T24 cells (5 × 10^6^ cells) suspended in 100 µL PBS were injected subcutaneously into the right flank of each mouse. Further experiments were performed after a 2-week or 5-month tumor growth period for the FaDu or T24 xenograft models. All animal manipulations were performed using sterile techniques.

### Animal experiments

For experiments using radio-high-performance liquid chromatography (HPLC), ^18^F-FMISO (338–825 MBq) was injected into the FaDu xenograft models via the tail vein; 4 h later, the mice were killed and tumor tissues immediately excised. Radio-HPLC study was performed with two mice. For IMS and autoradiography (ARG) experiments, a mixture of ^18^F-FMISO (10–33.5 MBq) and non-labeled FMISO (550 mg/kg; FutureChem Co., Ltd., Busan, Korea), dissolved in an aqueous solution (dimethylacetamide/saline = 1:3), was injected into the tumor-bearing mice via the tail vein. IMS and ARG study was performed with four or two mice for the FaDu or T24 tumor-bearing mice. At 2 h after FMISO injection, pimonidazole (100 mg/kg; Hypoxyprobe Inc., Burlington, MA, USA) was intravenously injected into the same mice. These mice were killed at 4 h after FMISO administration, and tumor tissues were immediately excised and frozen in dry ice powder. Serial cross sections of 10 µm thickness were immediately cut and thaw-mounted on a glass slide using a CM3050-Cryostat (Leica Microsystems, Wetzlar, Germany).

### Radio-HPLC analysis of tumor homogenates

The tumor of each mouse was weighed, suspended in PBS with protease inhibitor cocktail (4 mL/g of tissue; Roche Diagnostics, Basel, Switzerland) and crushed with zirconia beads using a Micro Smash instrument (Tomy Seiko Co., Ltd., Tokyo, Japan) at 4 °C. The homogenized samples were twice extracted with methanol. Concentrated extracts were chromatographed using a Shimadzu HPLC gradient system monitored at 220 nm (LC-20AD system; Shimadzu Corporation, Kyoto, Japan) equipped with an Atlantis HILIC column (150 mm × 4.6 mm, 5 µm; Waters Co., Milford, MA, USA), and eluted with a mobile phase composed of 5 mM ammonium formate (A) and acetonitrile/water (95:5) containing 5 mM ammonium formate (B). The analytes were eluted with a 90–50% B linear gradient. Consecutive 0.5 min HPLC fractions were collected during elution and the radioactivity of these fractions was measured with a gamma counter. Solutions of FMISO and amino-FMISO-GS gifted from Shionogi & Co., Ltd. (Toyonaka, Japan) were employed as standards.

### Autoradiography and immunohistochemical staining of pimonidazole

Autoradiographic images of tumor cryosections were acquired by a previously reported method [15]. Pimonidazole immunohistochemical staining of the serial tumor sections was performed to assess tumor hypoxia using the Hypoxyprobe-1 MAb1 antibody (Hypoxyprobe Inc.), as described previously [11].

### Sample preparation for MALDI-IMS

Tumor sections were placed on indium tin oxide-coated glass slides (Bruker Daltonics Inc., Billerica, MA, USA) and stored at −80 °C until analysis. Slides were placed in a vacuum desiccator for 15 min at room temperature and optical images were acquired using a scanner to identify the location of each tissue. Sections were then coated with the matrix solution (30 mg/mL 2,5-dihydroxybenzoic acid dissolved in a 1:1 v/v methanol–water solution containing 0.2% trifluoroacetic acid) with an ImagePrep automated device using vibrational vaporization technology (Bruker Daltonics Inc.).

### MALDI-IMS study

IMS analysis was performed with a 7T Bruker solariX XR MALDI Fourier transform-ion cyclotron resonance (FT-ICR MS; Bruker Daltonics Inc.) equipped with a SmartBeam II UV laser. Data were acquired and analyzed with flexImaging software (Bruker Daltonics Inc.). The laser energy and the raster step size were set at 40% and 150 µm, respectively. Analytes were detected in the positive-ion mode.

### LC–MS analysis

To obtain product ion spectra and to determine the concentration of amino-FMISO-GS, cell supernatants were injected into the LC–MS system. An UltiMate 3000 HPLC (Thermo Fisher Scientific Inc., Waltham, MA, USA) coupled to a Q Exactive Plus Hybrid Quadrupole-Orbitrap Mass Spectrometer (Thermo Fisher Scientific Inc.) was used for LC–MS analysis. Chromatographic separation was performed with a YMC-Triart C18 column (50 × 2 mm, 1.7 µm; YMC Co., Ltd., Kyoto, Japan), and analytes were eluted with a mobile phase composed of 5 mM ammonium hydrogen carbonate (A) and acetonitrile (B). The analytes were eluted with a 1–35% B linear gradient. ESI was performed in the positive-ion mode.

### Statistics

Data are represented as the mean ± SEM. Statistical analyses were performed with two-way ANOVA following the Tukey–Kramer test (for the cellular uptake study) or the Student’s *t* test (for other in vitro studies). JMP 11 software (SAS Institute Inc., Cary, NC, USA) was used for statistical analyses. A two-tailed value of *p* < 0.05 was considered to be statistically significant.

## Results

### In vitro studies

**Fig. 2:**
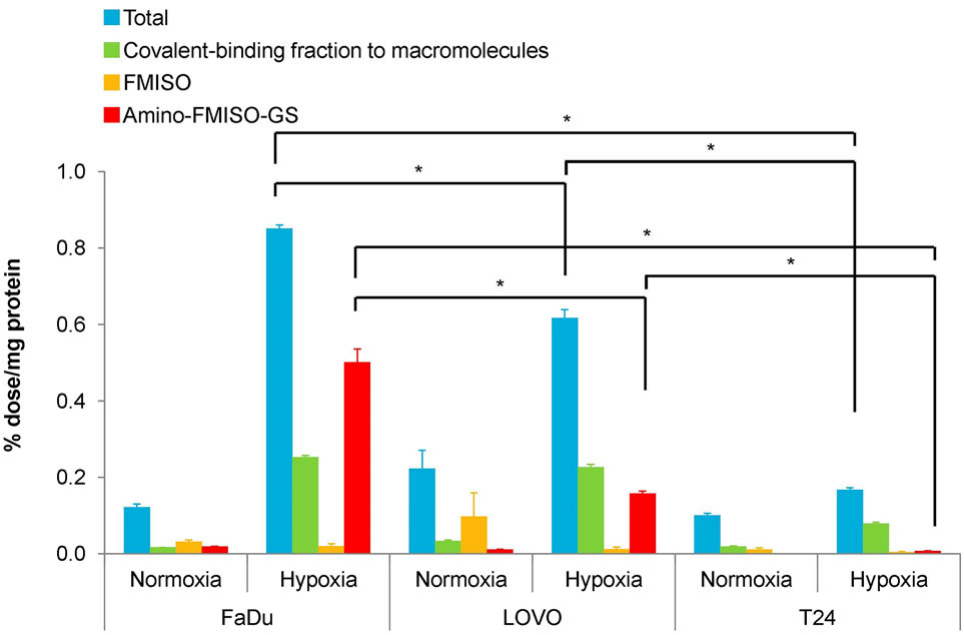
Cellular uptake and metabolism of FMISO with tumor cells in vitro. Cells were pre-incubated for 18 h under normoxic and hypoxic (1% O_2_) conditions. After addition of FMISO, the cells were incubated for 4 h under normoxic and hypoxic (1% O_2_) conditions. *p < 0.01

Under normoxic conditions, radioactivity uptake was low across all cell lines (FaDu; 0.122 ± 0.009, LOVO; 0.223 ± 0.048, T24; 0.100 ± 0.005% dose/mg protein; Fig. 2). In contrast, under hypoxic conditions, radioactivity uptake in FaDu cells reached 0.851 ± 0.009% dose/mg protein, followed by that in LOVO (0.617 ± 0.021% dose/mg protein), while that in T24 cells was only 0.167 ± 0.006% dose/mg protein. The distribution of radioactivity from FMISO covalently bound to macromolecules versus unbound FMISO was determined by methanol extraction to be 29.77 ± 0.47, 36.71 ± 1.28, and 47.20 ± 1.84% in FaDu, LOVO and T24 cells, respectively.

The presence of amino-FMISO-GS in the tumor cells was detected in all of cells with LC–MS. The amount of amino-FMISO-GS were higher in the same order as radioactivity uptake (0.502 ± 0.035 (FaDu; FMISO high-accumulating cells), 0.158 ± 0.006 (LOVO; FMISO medium-accumulating cells), and 0.007 ± 0.001 (T24; FMISO low-accumulating cells) % dose/mg protein, respectively).

### Cellular glutathione content and GST enzyme activity

**Fig. 3:**
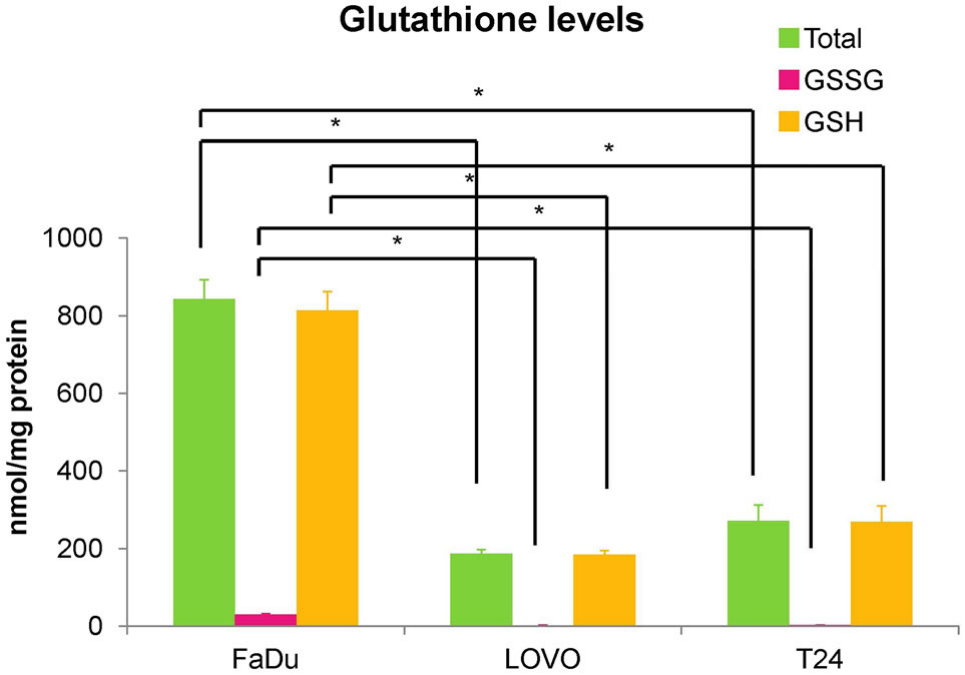
Cellular glutathione levels in tumor cells in vitro under hypoxic (1% O_2_) conditions. *p < 0.01

**Fig. 4:**
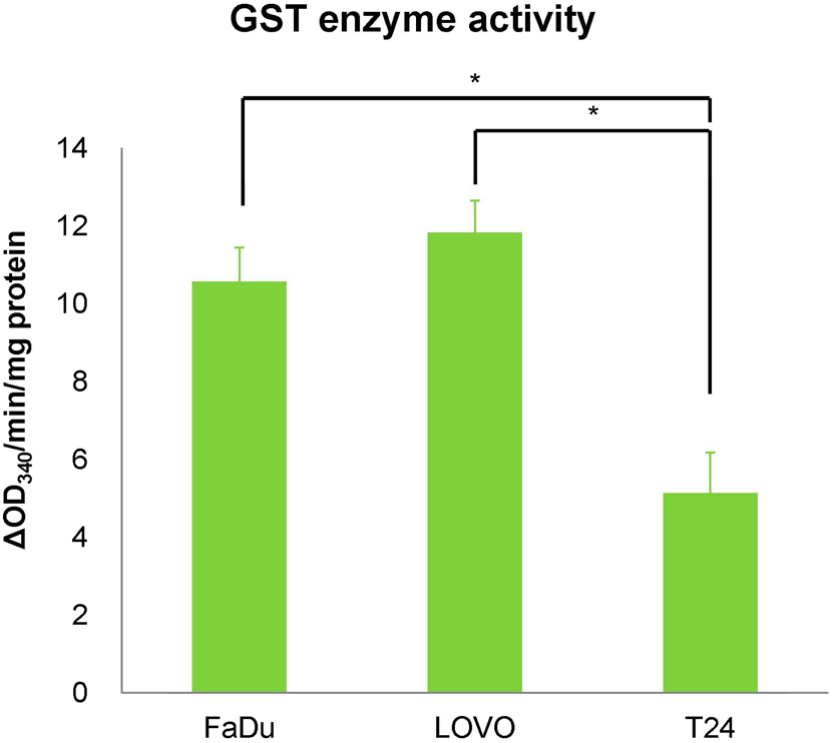
GST enzyme activity in tumor cells in vitro under hypoxic (1% O_2_) conditions. *p < 0.01

The intracellular glutathione levels and GST enzyme activity for the each of the cell lines studied are detailed in Figs. 3 and 4, respectively. In all cell lines, intracellular glutathione was found to be present mostly as GSH, which has the ability to bind to low-molecular-weight compounds to enhance their hydrophilicity. The GSH level in FaDu was the highest of the three cells, while those in LOVO and T24 were similar levels (650.5 ± 39.3 (FaDu), 147.7 ± 7.9 (LOVO) and 214.7 ± 32.7 (T24) nmol/mg protein, respectively). In addition, GST activity in FaDu was similar level to that in LOVO, while that in T24 was low (10.56 ± 0.87 (FaDu), 11.82 ± 0.83 (LOVO), and 5.13 ± 1.04 (T24) ΔOD_340_/min/mg protein, respectively).

### Expression of genes relating to glutathione conjugation

**Fig. 5:**
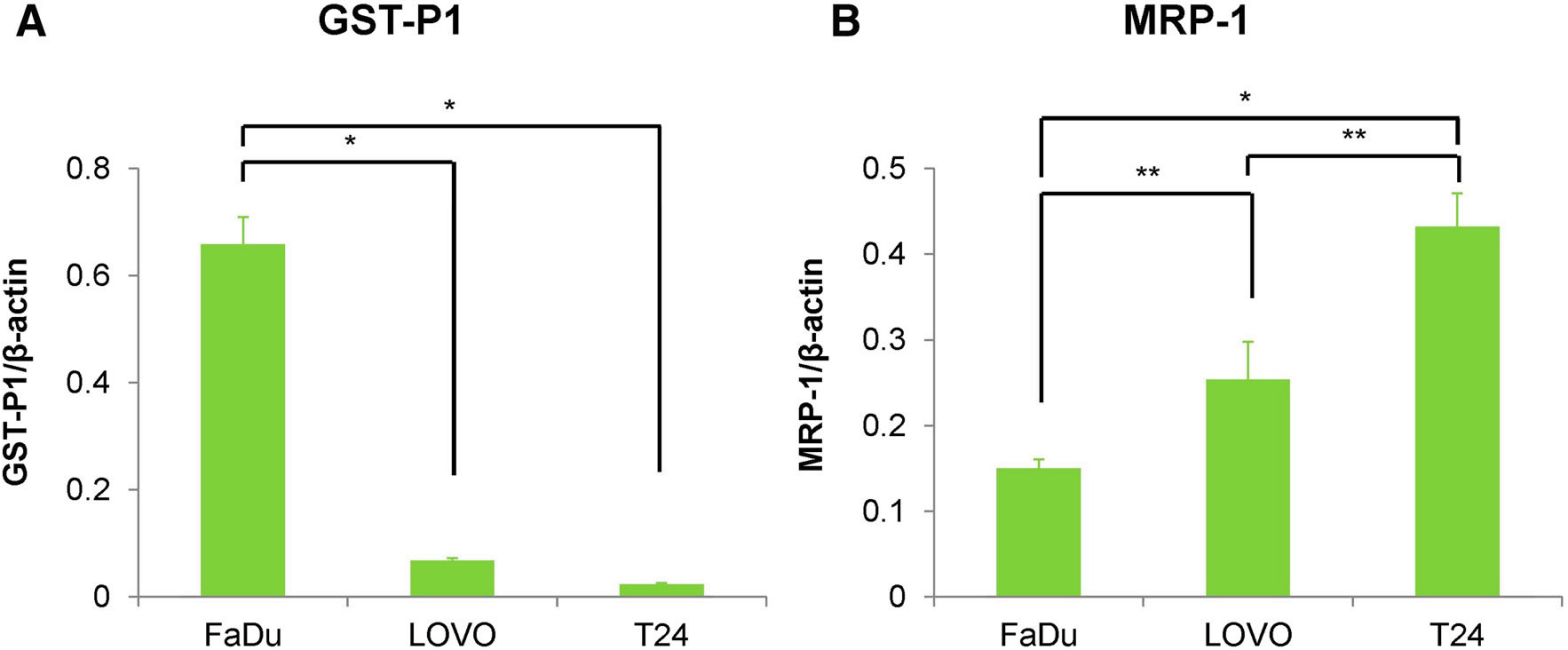
Quantitative RT-PCR analysis of mRNA expression levels in tumor cells under hypoxic (1% O_2_) conditions. **a** *GST*-*P1*. **b** *MRP*-*1*. *p < 0.01, **p < 0.05

The gene expression levels of *GST*-*P1* and multidrug resistance-associated proteins 1 (*MRP*-*1*) were evaluated with real-time PCR (Fig. 5). mRNA levels of the housekeeping gene β-actin served to normalize the mRNA levels across the experimental samples. Significantly higher expression of *GST*-*P1* was detected in the FaDu cells compared with the LOVO and T24 cells. Expression levels of *MRP*-*1* were in the inverse order (T24, LOVO, FaDu) with radioactivity uptake.

### Distribution of amino-FMISO-GS in tumors

**Fig. 6:**
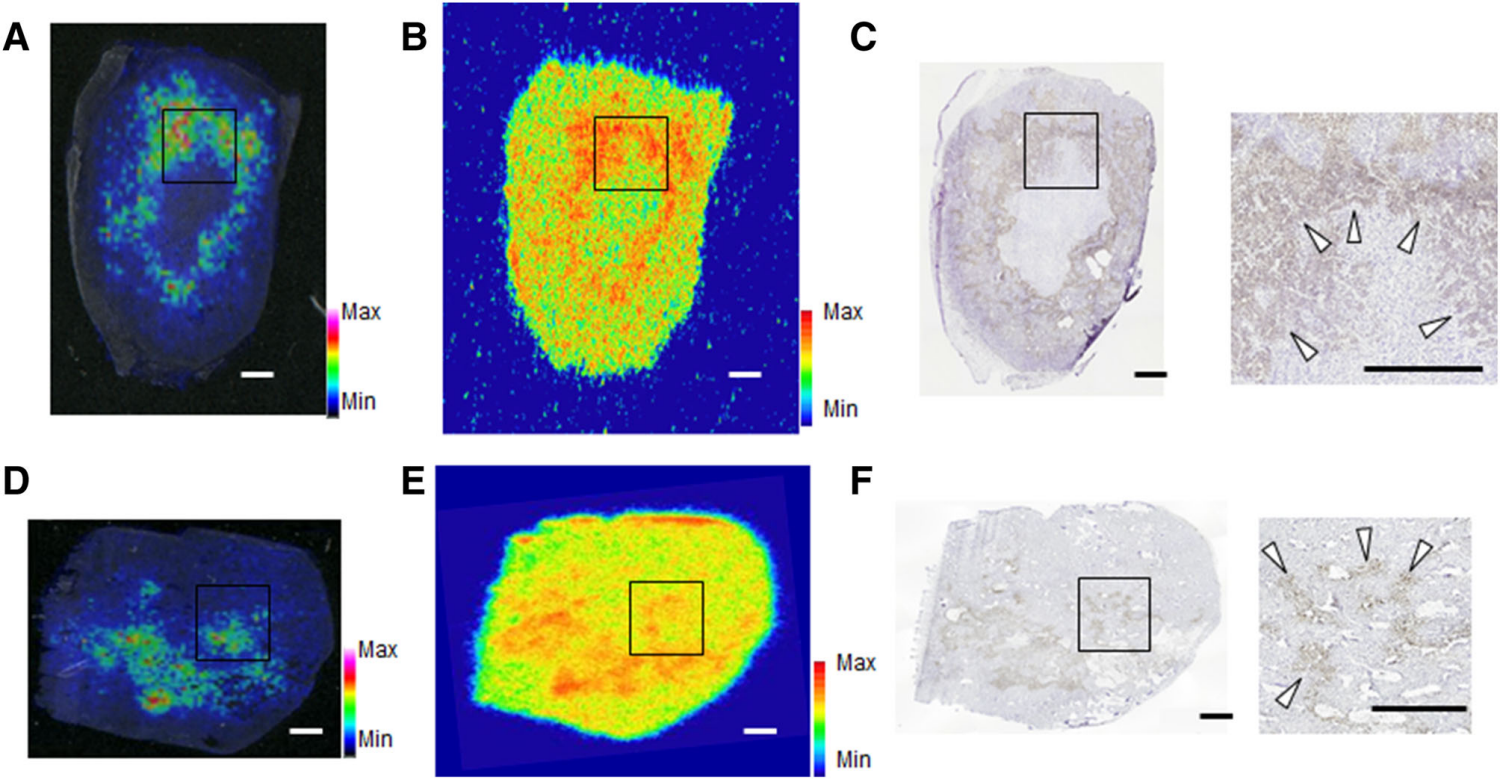
Distribution of the glutathione conjugate of amino-FMISO, illustrated by representative mass spectrometric images, ARG and pimonidazole staining in mouse tumors 4 h after administration of ^18^F-FMISO. *Scale bars* represent 1 mm. **a**–**c** Images of tumor cross section from FaDu-xenografted model mouse. **d**–**f** Images of tumor cross section from T24-xenografted model mouse. **a**, **d** Mass spectrometric images of m/z 465.157 exhibiting amino-FMISO-GS. **b**, **e** ARG images of a serial tumor section. **c**, **f** Immunohistochemical staining for pimonidazole. Details of the images at higher magnification are shown on the *right*. The areas of origin are highlighted in the original images

To evaluate the distribution of amino-FMISO-GS in tumor tissues, IMS analysis was performed. In both FaDu and T24 tumor-bearing mice, the distribution of amino-FMISO-GS (Fig. 6a, d) correlated with that observed with ARG (Fig. 6b, e) or positive pimonidazole immunohistochemical staining (Fig. 6c, f).

### Metabolite analysis of radiolabeled FMISO in tumor tissues

To characterize the FMISO metabolites, radio-HPLC analysis was performed in the FaDu xenograft model (Supplemental Fig. 1). The percentages of these two species were approximately 50 and 20% for amino-FMISO-GS and unmodified FMISO, respectively. Two peaks in the chromatogram were assigned to amino-FMISO-GS because it is proposed to consist of two isomers [16].

### Identification of amino-FMISO-GS in tumors

Using LC–MS analysis, the isotope pattern and MS/MS pattern of the synthetic and FaDu tumor homogenate samples were observed to correspond (Supplemental Fig. 2). Thus, the production of amino-FMISO-GS in tumors was verified and the structure was thought to be present in that of either or both of the 2 isomers [16, 17].

## Discussion

Our previous study found that the ^18^F-FMISO incorporated into hypoxic tumor cells was present as a glutathione conjugate of a reduced metabolite (amino-FMISO-GS) [11]. However, it was unclear whether the formation of this FMISO metabolite was occurred within hypoxic tumor cells and how this metabolite contributed to FMISO accumulation. Therefore, we first performed a cellular uptake study and LC–MS analysis to evaluate the levels of amino-FMISO-GS in FaDu, LOVO, and T24 cells. The accumulation levels of ^18^F-FMISO were significantly higher in hypoxic cells compared with normoxic cells independent of cell type, which is consistent with the current conventional understanding of hypoxia imaging with ^18^F-FMISO. Amino-FMISO-GS was found to be produced and its amount was significantly higher under hypoxic condition compared with normoxic condition in all FaDu, LOVO, and T24 cells (Fig. 2). These results indicated that amino-FMISO-GS was actually produced within hypoxic tumor cells. However, the amount of this conjugate as well as the total accumulation levels of ^18^F-FMISO varied according to the cell line under the same hypoxic conditions. Furthermore, amino-FMISO-GS was the main component of FMISO-derived compound in hypoxic FaDu cells (FMISO high-accumulating cells), which was also seen in the ex vivo metabolite analysis (Fig. 6, Supplemental Fig. 1).

In our cellular uptake study, the radioactivity uptake observed under hypoxic conditions was different among cell lines, despite the same levels of reduced oxygen being applied [18]. We therefore postulated that other factors also play a role in FMISO accumulation in hypoxic cells. As seen in our in vitro (Fig. 2) and in vivo experiments (Fig. 6, Supplemental Fig. 1), the FMISO incorporated into the hypoxic tumor cells was mainly metabolized to amino-FMISO-GS. Therefore, we hypothesized that the capacity for FMISO conjugation with glutathione may be a factor responsible for the different cellular uptake among the three cell lines. Thus, we evaluated several factors related to glutathione conjugation under hypoxic conditions [19].

Glutathione is a ubiquitous tripeptide in mammalian systems and is involved in detoxification. It exists in both reduced (GSH) and oxidized (GSSG) states [20]. GSH has the capacity to bind to low-molecular-weight compounds to enhance their hydrophilicity, while GSSG does not. The thiol group of GSH is known to conjugate to electrophilic xenobiotics in reactions catalyzed by GSTs, and GST enzyme activities constitute an important cellular protection mechanism against several types of cell damage arising from the mutagenic, carcinogenic, and toxic effects of these xenobiotic compounds. The GST family falls within a group of important phase II drug-metabolizing enzymes in the detoxification of many different xenobiotics in mammals [21]. Within the GST family, GSTπ is known to be the most abundant isoenzyme in various tumor cell lines, and humans have a single functional GSTπ gene termed *GST*-*P1* [22, 23]. Therefore, we next evaluated the GSH content, GST enzyme activity, and *GST*-*P1* expression levels in FaDu, LOVO, and T24 cells. The GSH level in FaDu was significantly higher compared to those in LOVO and T24, while those in LOVO and T24 showed similar levels. On the other hand, the levels of GSSG were very low in all cell types (Fig. 3). This result suggests that reactive glutathione (GSH) was more abundant in the FMISO high-accumulating (FaDu) cells. Furthermore, GST activity in FaDu was similar to that in LOVO, and those activities were higher compared to that in T24, which suggest that the glutathione conjugation reaction occurs at a higher rate in the FaDu and LOVO cells. These results indicated that cellular uptake of FMISO was affected not only by oxygen concentration but also cell’s ability of glutathione conjugation.

The MRPs belong to the ATP-binding cassette (ABC) transporter family and mediate the ATP-dependent export of many glutathione conjugates out of cells. MRP-1 expression has been reported to be correlated with resistance to the alkylating anti-cancer drug such as chlorambucil [24]. MRP-1 is known to be effective transporter of glutathione conjugates of a wide variety of substrates [25]; therefore, we also evaluated the mRNA expression levels of the transporter with quantitative RT-PCR. The expression level of *MRP*-*1* mRNA were in the inverse order with FMISO accumulation and highest in T24 cells (FMISO low-accumulating cells), followed by LOVO (FMISO medium-accumulating cells), and then FaDu (FMISO high-accumulating cells) (Fig. 5). It is possible that increased expression of MRP-1 promotes the efflux of amino-FMISO-GS outside of the cells, which may contribute to reduce the cellular accumulation levels of ^18^F-FMISO in the hypoxic cells.

Taken together, our results suggest that increased production and decreased excretion of amino-FMISO-GS in the FaDu cells contributed to the high accumulation of FMISO. FMISO is usually injected in a tracer dose and GSH levels in cells are in mM range, thus the intracellular GSH levels are much higher, compared with the levels required for the conjugation of FMISO. Therefore, GSH may relatively less contribute to FMISO accumulation among glutathione conjugation-related factors investigated in this study. The FMISO covalent binding ratio was higher in the inverse order with the amount of cellular amino-FMISO-GS and highest in T24, followed by LOVO, and then FaDu in the cellular uptake assay. These results suggest that formation of FMISO adducts to macromolecules occurred instead of glutathione conjugation in the cells with the lower glutathione conjugation ability such as T24.

Intracellular GSH and the processes of GSH conjugation and MRP-mediated efflux have been reported to synergistically influence resistance to chemotherapy, and phase II detoxification pathways including glutathione conjugation are known to serve as the basis for cellular resistance to cytotoxic drugs [24, 26, 27].

Therefore, it is likely that FMISO-PET depicts areas with high glutathione conjugation ability (high GSH content and GST activity and low MRP-1 expression) along with low oxygen levels. FMISO-PET images may therefore not provide a genuine reflection of tumor oxygen levels, as the imaging results appear to be influenced by glutathione conjugation ability in addition to oxygen levels. FMISO uptake may be determined by complicated factors, not any single factor. Intracellular GSH content, GST-mediated glutathione conjugation and MRP-mediated efflux might synergistically contribute to incorporation level of amino-FMISO-GS in cells. In addition, some anti-cancer drugs are known to form glutathione conjugates in cancer cells in the process of detoxification, and GSH depletion was reported in patients who received treatment with drugs such as cisplatin [26]; therefore, exposure to drugs that form glutathione conjugates may result in competition with FMISO accumulation in tumors by consuming intracellular GSH. These considerations suggest that FMISO accumulation and therefore FMISO-based imaging may be affected by tumor type or patient conditions such as treatment history.

In this study, we evaluated the uptake of the ^18^F-FMISO probe in three cell lines, and evaluated the effect of factors such as oxygen levels and glutathione conjugation capacity. Although further detailed studies would be required such as the evaluation of FMISO uptake under blocking GSTπ and MRP-1, we have illustrated the possibility that FMISO accumulation is correlated with glutathione conjugation ability, and these findings need to be taken into account in future FMISO-based imaging studies. In conclusions, our study suggests that FMISO accumulates in hypoxic cells through reductive metabolism followed by glutathione conjugation. Factors favoring increased formation and limited efflux of the glutathione conjugate of amino-FMISO may contribute significantly to the accumulation of FMISO in the cells, along with hypoxic conditions.

## Electronic supplementary material

Below is the link to the electronic supplementary material.

Supplemental Fig. 1 Radio-HPLC chromatogram of the low-molecular-weight fraction of FMISO, illustrating the relative proportions of unmodified FMISO and amino-FMISO-GS (JPEG 228 kb)

Supplemental Fig. 2 Validation of amino-FMISO-GS in mouse tumors through the analysis of isotope and MS/MS patterns. A: Structure and predicted MS/MS pattern of amino-FMISO-GS. B: Isotope pattern of amino-FMISO-GS observed for the synthetic form and from that obtained from a mouse tumor. C: Fragment pattern from MS/MS analysis of ion m/z 465.157 in a mouse tumor (JPEG 349 kb)

Supplementary material 3 (DOCX 17 kb)
