# Supplementary figures and images for: FMISO accumulation in tumor is dependent on glutathione conjugation capacity in addition to hypoxic state
Source: Ann Nucl Med. 2017 Jul 10;31(8):596–604. doi: 10.1007/s12149-017-1189-9 (PMC5622914; doi:10.1007/s12149-017-1189-9)

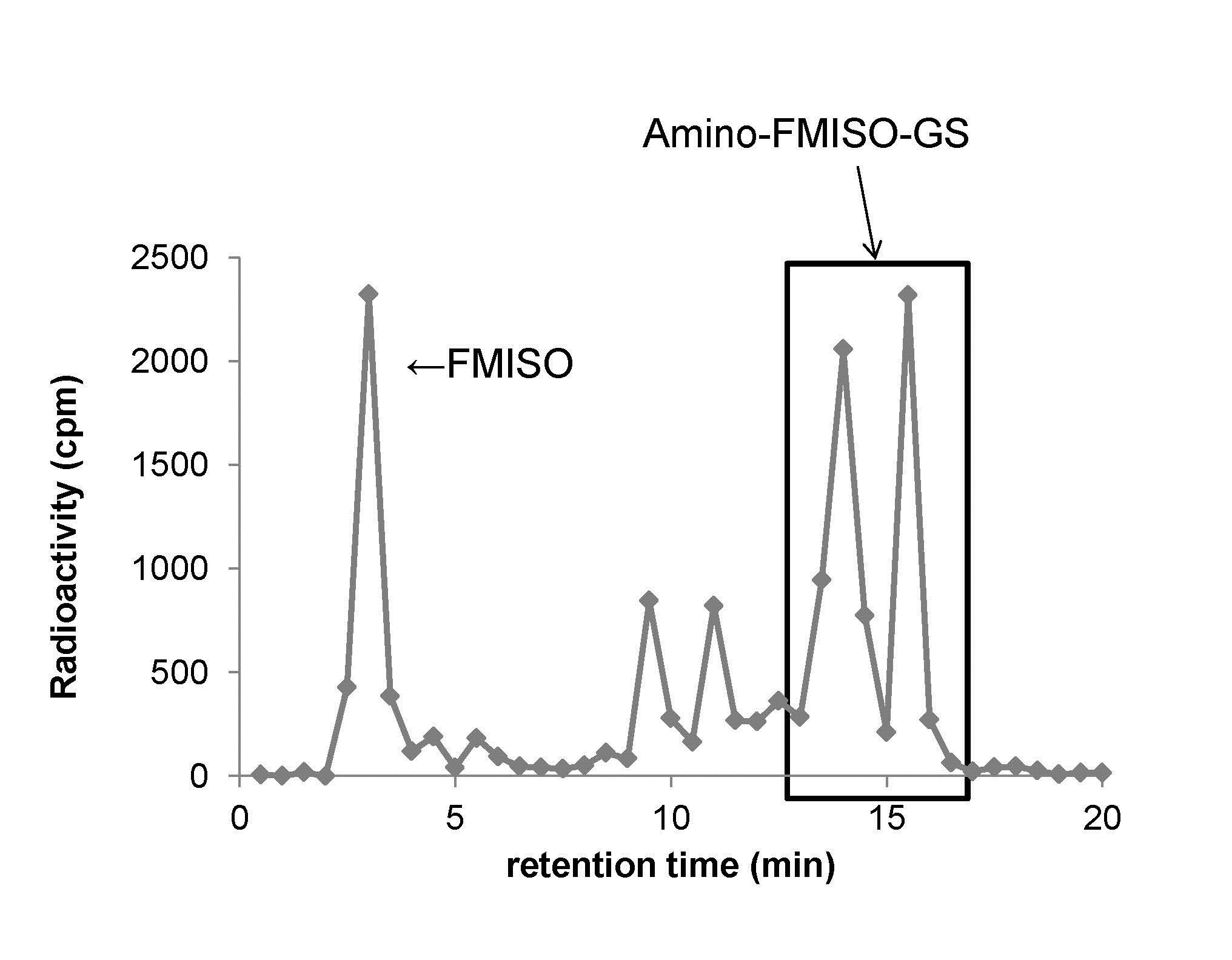

Supplement: Supplementary file 1 — Supplemental Fig. 1 Radio-HPLC chromatogram of the low-molecular-weight fraction of FMISO, illustrating the relative proportions of unmodified FMISO and amino-FMISO-GS (JPEG 228 kb) [file 12149_2017_1189_MOESM1_ESM.jpg]

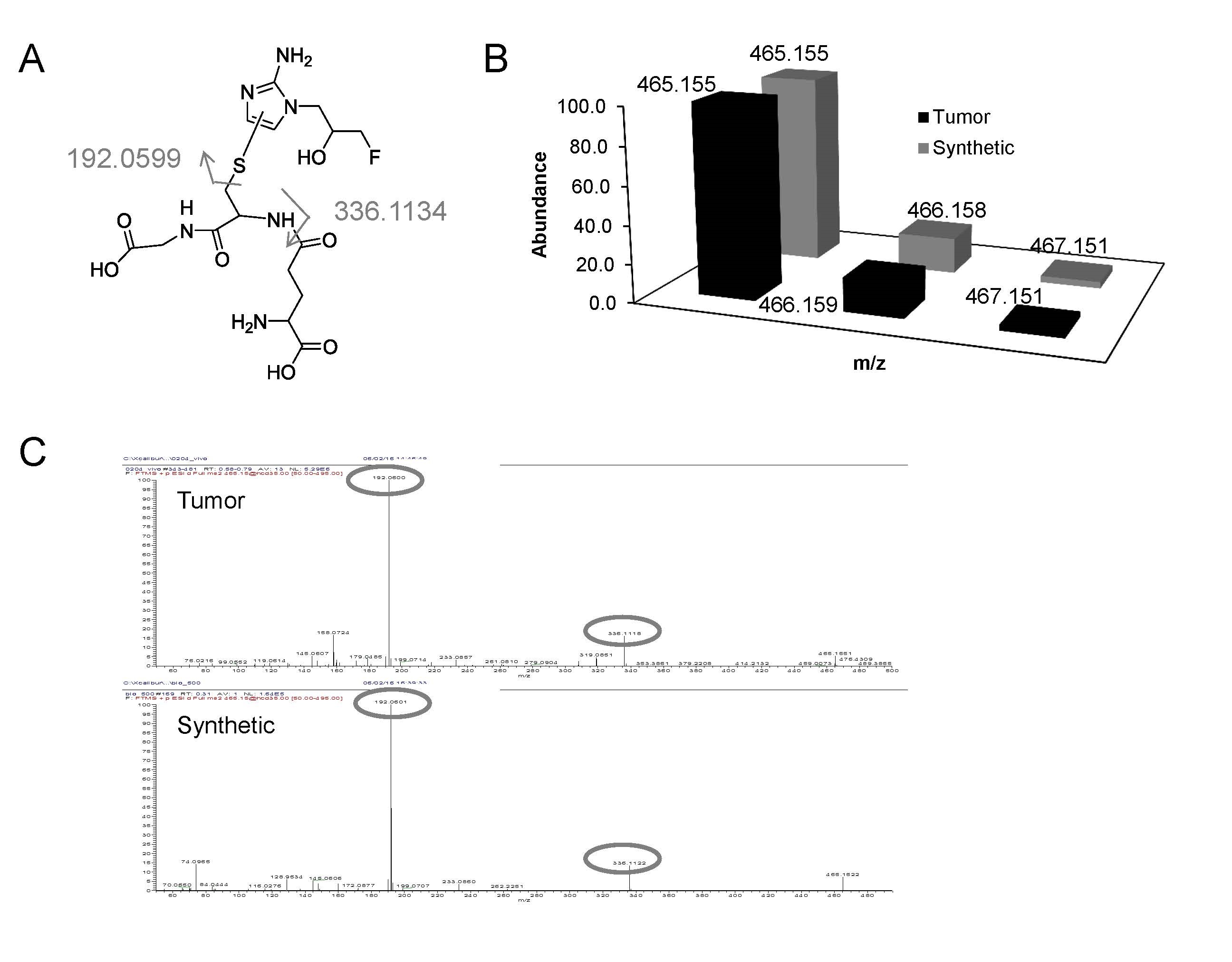

Supplement: Supplementary file 2 — Supplemental Fig. 2 Validation of amino-FMISO-GS in mouse tumors through the analysis of isotope and MS/MS patterns. A: Structure and predicted MS/MS pattern of amino-FMISO-GS. B: Isotope pattern of amino-FMISO-GS observed for the synthetic form and from that obtained from a mouse tumor. C: Fragment pattern from MS/MS analysis of ion m/z 465.157 in a mouse tumor (JPEG 349 kb) [file 12149_2017_1189_MOESM2_ESM.jpg]
