# Supplementary material for: FMISO accumulation in tumor is dependent on glutathione conjugation capacity in addition to hypoxic state
Source: Ann Nucl Med. 2017 Jul 10;31(8):596–604. doi: 10.1007/s12149-017-1189-9 (PMC5622914; doi:10.1007/s12149-017-1189-9)
Supplement: Supplementary file 3 — Supplementary material 3 (DOCX 17 kb) [file 12149_2017_1189_MOESM3_ESM.docx]

Supplemental Table 1. Summary of primer sets used for quantitative real-time PCR.

| Primers | Sequences 5′–3′ | Reference* |
| --- | --- | --- |
| β-actin forward | AGGAAGGAAGGCTGGAAGAG | (*1*) |
| β-actin reverse | GGAAATCGTGCGTGACATTA | (*1*) |
| GSTP1 forward | ATGACTATGTGAAGGCACTG | (*2*) |
| GSTP1 reverse | AGGAAGGAAGGCTGGAAGAG | (*2*) |
| MRP-1 forward | CCCTGCACTGTCCGTCAC | (*3*) |
| MRP-1 reverse | CTGAGTTCCTGCGTACCTAT | (*3*) |

*References

1. Ben Amara A, Gorvel L, Baulan K, Derain-Court J, Buffat C, Verollet C, et al. Placental macrophages are impaired in chorioamnionitis, an infectious pathology of the placenta. J Immunol. 2013 Dec 1;191(11):5501-14.

2. Li T, Zhao XP, Wang LY, Gao S, Zhao J, Fan YC, et al. Glutathione S-transferase P1 correlated with oxidative stress in hepatocellular carcinoma. Int J Med Sci. 2013;10(6):683-90.

3. Chen L, Feng P, Li S, Long D, Cheng J, Lu Y, et al. Effect of hypoxia-inducible factor-1alpha silencing on the sensitivity of human brain glioma cells to doxorubicin and etoposide. Neurochem Res. 2009 May;34(5):984-90.
